# Anticollagen type II antibodies are associated with an acute onset rheumatoid arthritis phenotype and prognosticate lower degree of inflammation during 5 years follow-up

**DOI:** 10.1136/annrheumdis-2016-210873

**Published:** 2017-03-23

**Authors:** Vivek Anand Manivel, Mohammed Mullazehi, Leonid Padyukov, Helga Westerlind, Lars Klareskog, Lars Alfredsson, Saedis Saevarsdottir, Johan Rönnelid

**Affiliations:** 1 Department of Immunology, Genetics and Pathology, Uppsala University, Uppsala Sweden; 2 Rheumatology Unit, Department of Medicine, Karolinska University Hospital and Karolinska Institutet, Stockholm, Sweden; 3 Institute of Environmental Medicine, Karolinska Institutet, Stockholm, Sweden

**Keywords:** Rheumatoid Arthritis, DAS28, Outcomes research

## Abstract

**Objective:**

Antifibrillar collagen type II (anti-CII) antibody-positive patients with rheumatoid arthritis (RA) have early but not late signs of increased inflammation and joint erosions. We wanted to replicate this in a large RA cohort, and to relate to human leukocyte antigen (HLA)-DRB1* alleles.

**Methods:**

Anti-CII and anti-cyclic citrullinated peptide (CCP)2 were measured at baseline in 773 patients with RA from the Swedish Epidemiological Investigation in Rheumatoid Arthritis (EIRA) study with clinical follow-up data from the Swedish Rheumatology Quality Register (SRQ) registry, and 1476 with HLA-DRB1* information. Comparisons were done concerning C reactive protein (CRP), erythrocyte sedimentation rate (ESR), tender joint count (TJC), swollen joint count (SJC), Disease Activity Score encompassing 28 joints based on ESR (DAS28), DAS28CRP, pain-Visual Analogue Scale (VAS), global-VAS and Health Assessment Questionnaire Score (HAQ) at eight occasions during 5 years, and association with HLA-DRB1* alleles.

**Results:**

Anti-CII associated with elevated CRP, ESR, SJC, DAS28 and DAS28CRP at diagnosis and up to 6 months, whereas anti-CCP2 associated with SJC and DAS28 from 6 months to 5 years, but not earlier. The anti-CII-associated phenotype was strong, and predominated in anti-CII/anti-CCP2 double-positive patients. Anti-CII was associated with improvements in CRP, ESR, SJC, TJC and DAS28, whereas anti-CCP2 was associated with deteriorations in SJC and DAS28 over time. Anti-CII-positive patients achieved European League Against Rheumatism good or moderate response more often than negative patients. Anti-CII was positively associated with HLA-DRB1*01 and HLA-DRB1*03, with significant interaction, and double-positive individuals had >14 times higher mean anti-CII levels than HLA double negatives. Whereas smoking was associated with elevated anti-CCP2 levels, smokers had lower anti-CII levels.

**Conclusions:**

Anti-CII seropositive RA represents a distinct phenotype, in many respects representing the converse to the clinical, genetic and smoking associations described for anticitrullinated protein peptide autoantibodies. Although not diagnostically useful, early anti-CII determinations predict favourable inflammatory outcome in RA.

## Introduction

Rheumatoid arthritis (RA) is a multifactorial disease. RA can be classified as seropositive by the presence of rheumatoid factor and/or anticitrullinated protein peptide autoantibodies (ACPA).^1^ ACPA-positive RA represents a distinct phenotype associated with genetic and environmental factors, notably the HLA-DRB1* shared epitope (SE) and smoking.^2^ ^3^ The fibrillar collagen type II (CII) is essentially restricted to hyaline cartilage, where it is the major protein.^4^ A subgroup of patients with RA (3%–27%) have elevated levels of antibodies against CII (anti-CII), especially around the time of RA diagnosis, whereafter levels decline.^5–7^ We have described that anti-CII bound to CII in surface-bound immune complexes (IC) can induce pro-inflammatory cytokines and chemokines from mononuclear cells (MNC) and polymorphonuclear granulocytes (PMN).^8–10^ Anti-CII are thus functionally active, and we have previously shown that changes in anti-CII levels temporally associate with in vitro function of anti-CII-containing IC and to C reactive protein (CRP) and erythrocyte sedimentation rate (ESR) in corresponding serum samples. Anti-CII thus represent a RA phenotype with early but not late signs of inflammation.^9^ ^11^ This is in contrast to ACPA, associated with late occurrence of signs and symptoms of inflammation in the same RA cohort.^12^

This previous comparison of the anti-CII-dependent and ACPA-dependent RA phenotypes was performed in a small group of patients (n=274). By linking the Swedish Epidemiological Investigation in Rheumatoid Arthritis (EIRA) study to the Swedish Rheumatology Quality Register (SRQ), we have obtained clinical follow-up data in a larger RA cohort. Here, we validate and extend the characterisation of the anti-CII-dependent acute onset RA phenotype, and show that it also represents the contrariety to the ACPA-associated phenotype concerning association with HLA-DRB1* and smoking.

## Patients and methods

### Study subjects

EIRA patients (n=2000) and controls (n=960) were included between 1996 and 2005. All patients fulfilled the 1987 American College of Rheumatology classification criteria.^13^ Controls were selected from the Swedish population register and matched for age, locality and sex. Detailed description of EIRA and the clinical follow-up data acquired through linkage to SRQ has been described previously.^14–16^ All participants consented to join the study that was approved by the ethical committee of Karolinska Institutet.

SRQ data included CRP, ESR, swollen joint count (SJC), tender joint count (TJC), Disease Activity Score encompassing 28 joints based on ESR (DAS28) or CRP (DAS28CRP), Visual Analogue Scale data for pain (pain-VAS) and global disease activity (global-VAS) and Health Assessment Questionnaire Score (HAQ). Exclusion was made of patients lacking ACPA data (n=18), disease duration >365 days at diagnosis (n=170), patients lacking linked SRQ data (n=650), >10 days between clinical diagnosis and inclusion in EIRA (n=226) and non-specific anti-CII reactivity (n=163). Of the remaining 773 patients, SRQ data were available for 768 (99.4%) at baseline, 663 (85.8%) at 3 months, 627 (81.1%) at 6 months, 725 (93.8%) at 1 year, 669 (86.6%) at 2 years, 426 (55.1%) at 3 years, 265 (34.3%) at 4 years and 480 (62.1%) at 5 years.

HLA association studies were performed in 1476 patients, after exclusion of patients lacking information on anti-CCP2 (n=18) or HLA-DRB1* (n=23), disease duration >365 days (n=163) or non-specific reactivity (n=316).

### Detection of anti-CII antibodies

Anti-CII antibodies were measured as previously described by ELISA using human native collagen type II (Chondrex, Redmond, Washington, USA) as antigen. Levels >95th percentile of blood donors (29 AU/mL) were considered positive.^11^ Serum samples yielding higher optical density (OD) in blocked wells without the CII antigen were regarded as non-specific, and were treated separately.

### Anti-CCP2 measurements, genotyping and smoking data

Anti-CCP2 was measured by ELISA (Immunoscan CCPlus, Euro-Diagnostica, Malmö, Sweden) with a cut-off of 25 U/mL. Genotyping was done by PCR using sequence-specific primers. HLA-DRB1* alleles 0101/0401/0404/0405/0408/10 were defined as SE as described previously.^17–19^ Patients were classified as ever or never smokers.

### Statistical analysis

Associations between anti-CII and anti-CCP2 and clinical and laboratory measures were performed with the Mann-Whitney U test. Two-way analysis of variance (ANOVA) was used to study the association between anti-CII and anti-CCP2 status and clinical and laboratory measures, as well as between anti-CII levels and HLA-DRB1*01 and HLA-DRB1*03 alleles. ORs between HLA-DRB1 alleles and anti-CII and anti-CCP2 status and attainment of European League Against Rheumatism (EULAR) response (good and moderate compared with no response) were calculated with 95% CIs. The impact of age, sex and smoking status were investigated with logistic regression, but had only minor impact and were left out in the final calculations. In some comparisons with HLA status, a higher anti-CII cut-off corresponding to tumour necrosis factor induction by the corresponding IC in vitro were used.^9^ ^20^ HLA associations were investigated for all individuals and after excluding of SE-positive patients, as ACPA are strongly linked to SE. All statistical analyses were done using JMP11.

## Results

### Anti-CII and anti-CCP2 antibodies in EIRA

Among 1476 patients, 97 (6.6%) were anti-CII positive and 855 (57.9%) were anti-CCP2 positive. Thirty-nine patients (2.6%) had only anti-CII, 797 (54%) had only anti-CCP2, 58 (3.9%) were double positive and 582 (39.4%) lacked both antibodies. Among the EIRA controls, 15/926 (1.6%) were anti-CII positive (34 showed non-specific binding) and 16/958 (1.7%) were anti-CCP2 positive. Anti-CII levels were significantly higher among patients than among controls (median (mean) 13.3 (38.4) vs 9.3 (21.6) AU/mL, p<0.0001). There was no association between the occurrence of anti-CII and anti-CCP2 among patients (p=0.7), nor between anti-CII levels and age. The baseline disease-modifying antirheumatic drug (DMARD) usage did not differ between patients with and without anti-CII and anti-CCP2, respectively.

### Anti-CII and anti-CCP2 associations with clinical and laboratory measures

**Figure 1:**
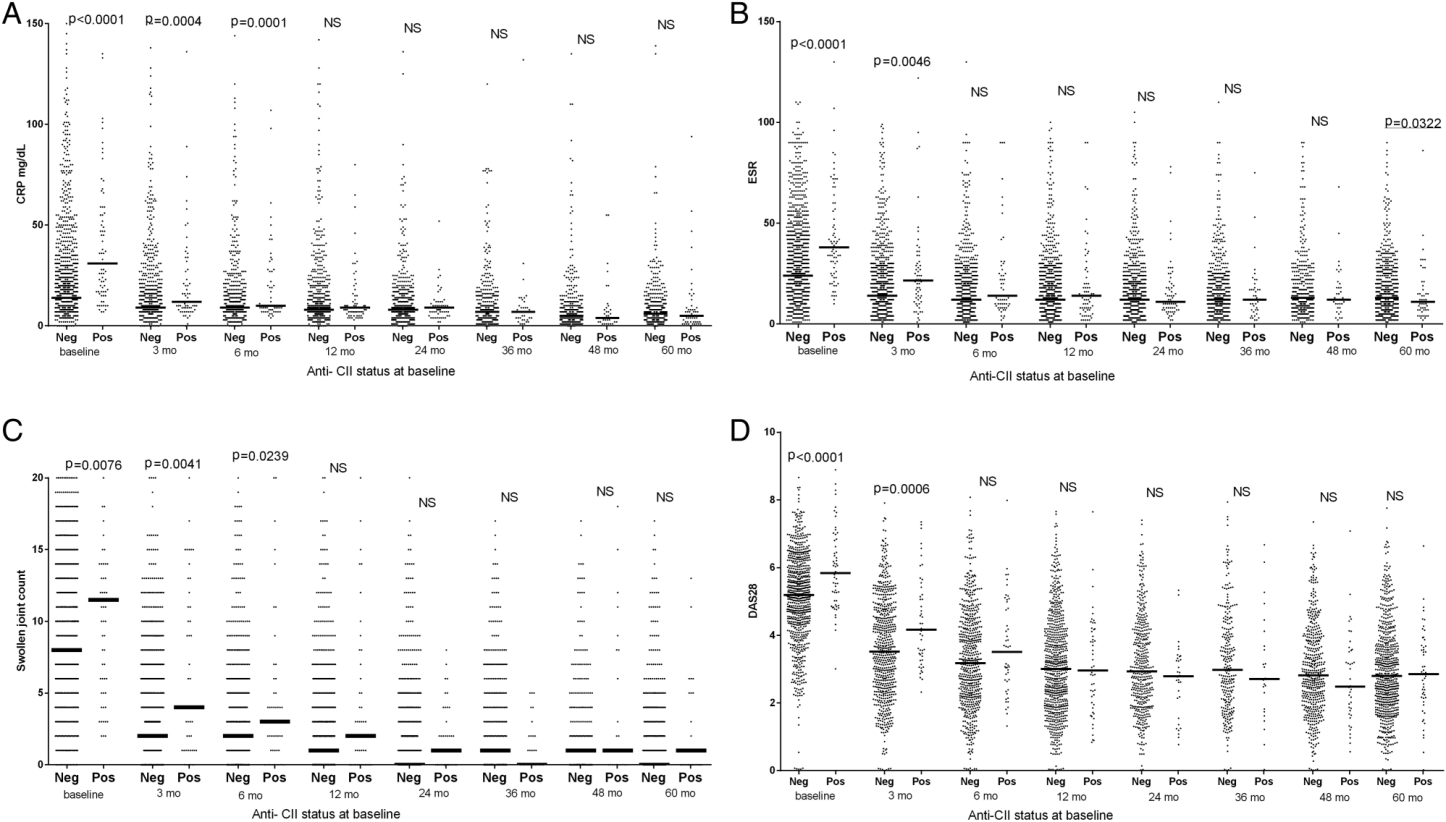
Association between baseline antifibrillar collagen type II (anti-CII) levels and (A) C reactive protein (CRP), (B) erythrocyte sedimentation rate (ESR), (C) swollen joint count (SJC) and (D) Disease Activity Score encompassing 28 joints based on ESR (DAS28) during 5 years follow-up in 773 newly diagnosed patients with rheumatoid arthritis (RA). Figures show significance between anti-CII-positive and anti-CII-negative patients at the different time points; only significant (p<0.05) differences are shown. The underlined p value for ESR after 5 years indicate lower median levels in the initially anti-CII-positive group. Data on the same patients dichotomised according to anti-CCP status are shown in figure 2. Data on (A) 29 and (C) 57 individuals with very high values were not depicted in the graphs, but were included in the statistical calculations. mo, months; neg, negative; NS, not significant; pos, positive.

**Figure 2:**
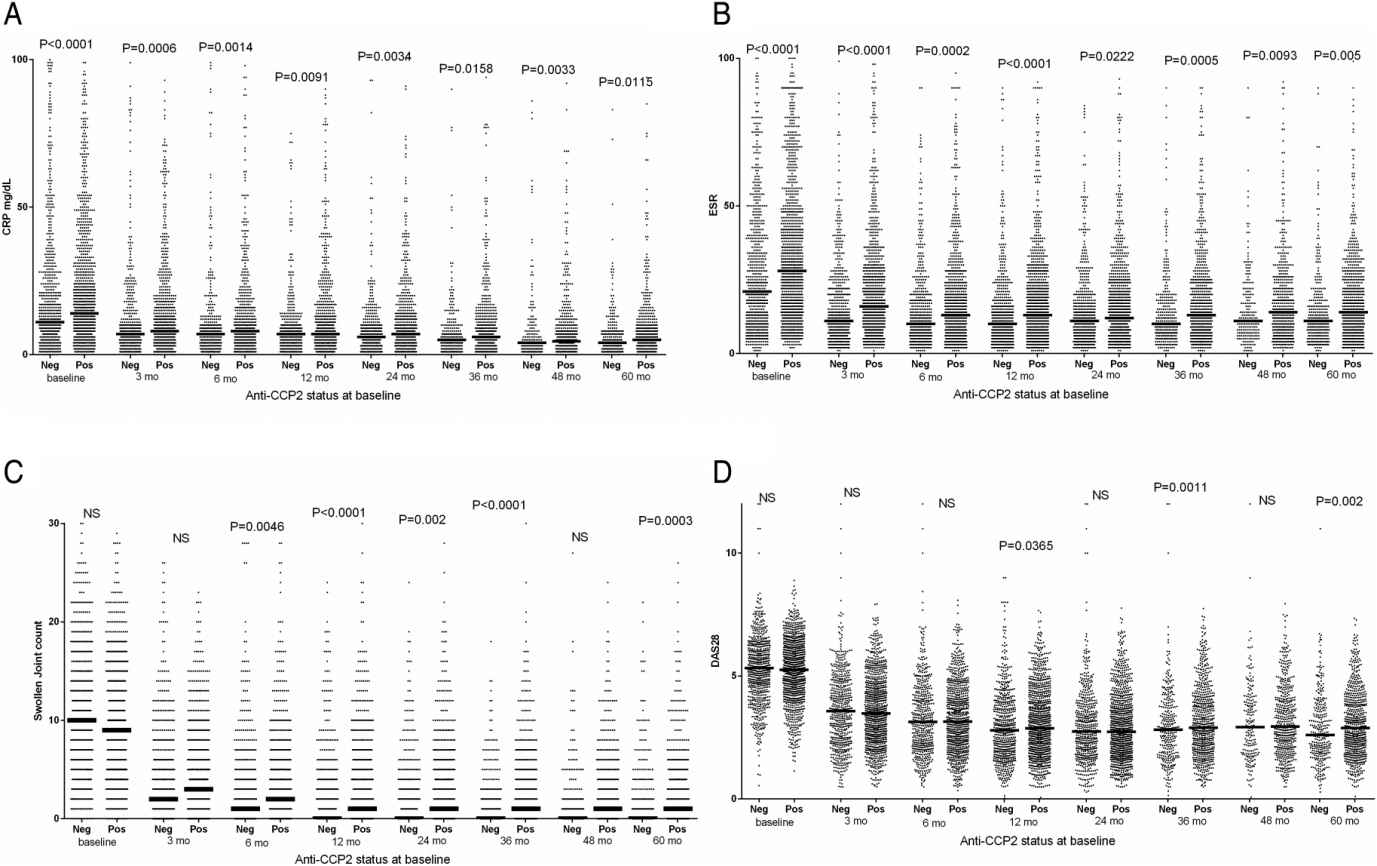
Association between baseline anti-CCP levels and (A) C reactive protein (CRP), (B) erythrocyte sedimentation rate (ESR), (C) swollen joint count (SJC) and (D) Disease Activity Score encompassing 28 joints based on ESR (DAS28) during 5 years follow-up in 773 newly diagnosed patients with rheumatoid arthritis (RA). Figures show significance between anti-CCP-positive and anti-CCP-negative patients at the different time points; only significant (p<0.05) differences are shown. Data on the same patients dichotomised according to antifibrillar collagen type II status are shown in figure 1. Data on (A) 122, (B) 21, (C) 52 and (D) 58 individuals with very high values were not depicted in the graphs, but were included in the statistical calculations. mo, months; neg, negative; NS, not significant; pos, positive.

As illustrated in figures 1 and 2 and online supplementary figures S1 and S2, the occurrence of anti-CII was associated with higher CRP values at baseline and the first follow-up visits; and the same was evident for ESR, SJC, DAS28 and DAS28CRP. Anti-CCP2 on the other hand was associated with higher disease activity later during the 5-year follow-up, and with CRP and ESR during the full period.

10.1136/annrheumdis-2016-210873.supp1 supplementary data

In early time points when anti-CII-positive patients showed elevated activity measures, non-specific samples showed values between negative and positive samples, usually significantly lower than anti-CII-positive individuals (see online supplementary table S1). The temporal association between anti-CII levels and early inflammation was strong: we originally chose patients with up to 40 days between clinical phenotype (SRQ data) and antibody measurement (EIRA inclusion), but every significant association with early inflammation became stronger when we restricted the time difference to 10 days (data not shown).

The 773 patients were divided into patients expressing only anti-CII (n=20), only anti-CCP2 (n=432) or both (n=36) and each group was compared with double-negative patients (n=285). Patient expressing only anti-CII or only anti-CCP mirrored the phenotypes described above, anti-CII was associated with high measures for CRP, ESR, SJC, DAS28 and DAS28CRP and HAQ early, whereas anti-CCP2 was associated with high measures for SJC, TJC, DAS28, DAS28CRP late, and with CRP and ESR during the whole follow-up period. Patients in the anti-CII and anti-CCP2 double-positive group mainly followed the anti-CII pattern, with early but not late increased values for CRP, ESR, DAS28 and DAS28CRP, but with a mixed pattern for SJC (see table 1 and online supplementary table S2).

**Table 1:** Associations between the occurrence of anti-CII and anti-CCP, individually or in combination and clinical symptoms during 5-year follow-up after RA diagnosis

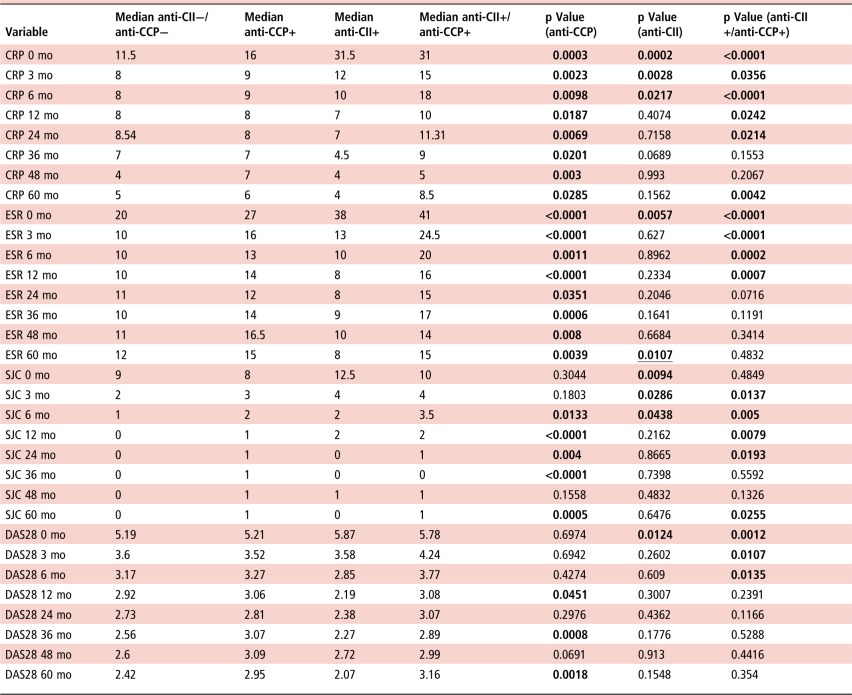

Median levels are shown for anti-CII/anti-CCP double-negative patients (n=285), anti-CII-positive and anti-CCP-negative patients (n=20), anti-CII-negative and anti-CCP-positive patients (n=432) and anti-CII-positive and anti-CCP2-positive patients (n=36). p Values refer to comparisons with the anti-CII-negative/anti-CCP2 double-negative group. Significant differences are depicted in bold, and also underlined if the median level for the corresponding antibody is lower than for the double-negative group.

Corresponding data for tender joint count, DAS28CRP, pain-VAS, global-VAS and Health Assessment Questionnaire are shown in online supplementary table S2.

CII, collagen type II; CRP, C reactive protein; DAS28, Disease Activity Score encompassing 28 joints based on ESR; ESR, erythrocyte sedimentation rate; mo, months; RA, rheumatoid arthritis; SJC, swollen joint count; VAS, Visual Analogue Scale.

The ANOVA analyses confirmed that anti-CII was associated with CRP, ESR, SJC, DAS28, DAS28CRP and HAQ early, whereas anti-CCP2 was associated with late elevations CRP, SJC, DAS28 and DAS28CRP, and with ESR during all time points except 48 months. Except for concerning ESR, anti-CII and anti-CCP2 showed marginal interactions (see online supplementary table S3).

### Anti-CII and anti-CCP associations with changes in clinical and laboratory measures

When the occurrence of anti-CII and anti-CCP2 were associated with changes as compared with corresponding baseline measures, anti-CII was associated with most, especially late changes in CRP, ESR, SJC, TJC, DAS28, DAS29CRP, but also with changes in pain-VAS, global-VAS and HAQ. Anti-CCP2 was associated with fewer changes: in ESR until 48 months, SJC until 12, 24, 36 and 60 months, TJC until 36 months, DAS28 until 36 months, DAS28CRP until 12, 24 and 36 months and HAQ until 36 months. In all cases, significant changes for anti-CII were associated with larger improvements than for antibody double-negative subjects, and all significant changes for anti-CCP2 except ESR at 48 months and HAQ at 36 months were associated with smaller improvements as compared with antibody double-negative subjects (see table 2 and online supplementary table S4).

**Table 2:** Association between changes in inflammatory markers as compared with baseline values and the occurrence of anti-CII and anti-CCP2 at the time of RA diagnosis

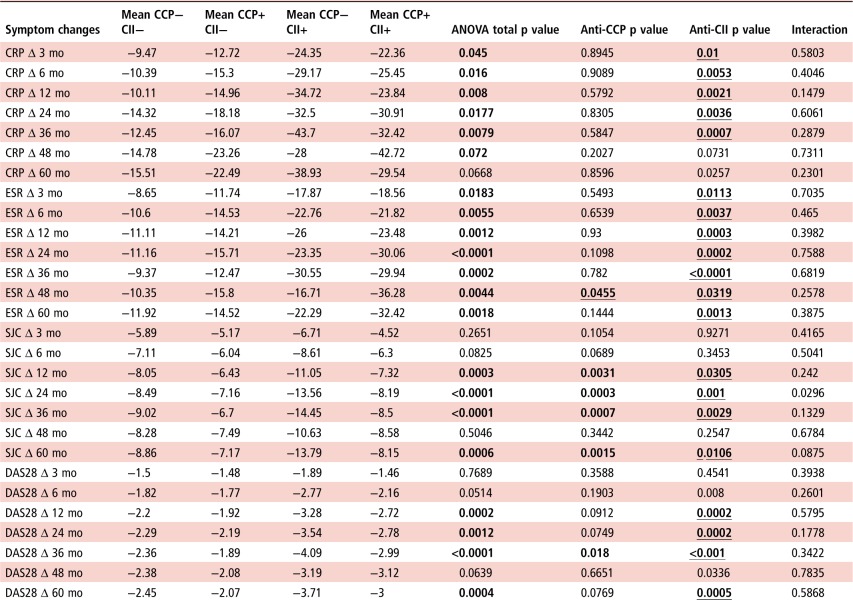

Analysis was performed with two-way ANOVA, and changes in clinical and laboratory measures were expressed as differences between values at different time points and corresponding baseline values. Mean levels are shown for anti-CII/anti-CCP double-negative subjects (n=285), anti-CII-positive and anti-CCP-negative subjects (n=20), anti-CII-negative and anti-CCP-positive patients (n=432) and anti-CII-positive anti-CCP2-positive patients (n=36). p Values for the total ANOVA, anti-CCP2, anti-CII and the interaction between anti-CII and anti-CCP2 are given in individual columns. Significant p values for the individual antibodies are depicted in bold, and also underlined if the mean level for the corresponding antibody is lower than for the double-negative group. Corresponding data for tender joint count, DAS28CRP, pain-VAS, global-VAS and Health Assessment Questionnaire are shown in online supplementary table S2.

ANOVA, analysis of variance; CII, collagen type II; CRP, C reactive protein; DAS28, Disease Activity Score encompassing 28 joints based on ESR; ESR, erythrocyte sedimentation rate; mo, months; RA, rheumatoid arthritis; SJC, swollen joint count; VAS, Visual Analogue Scale.

**Figure 3:**
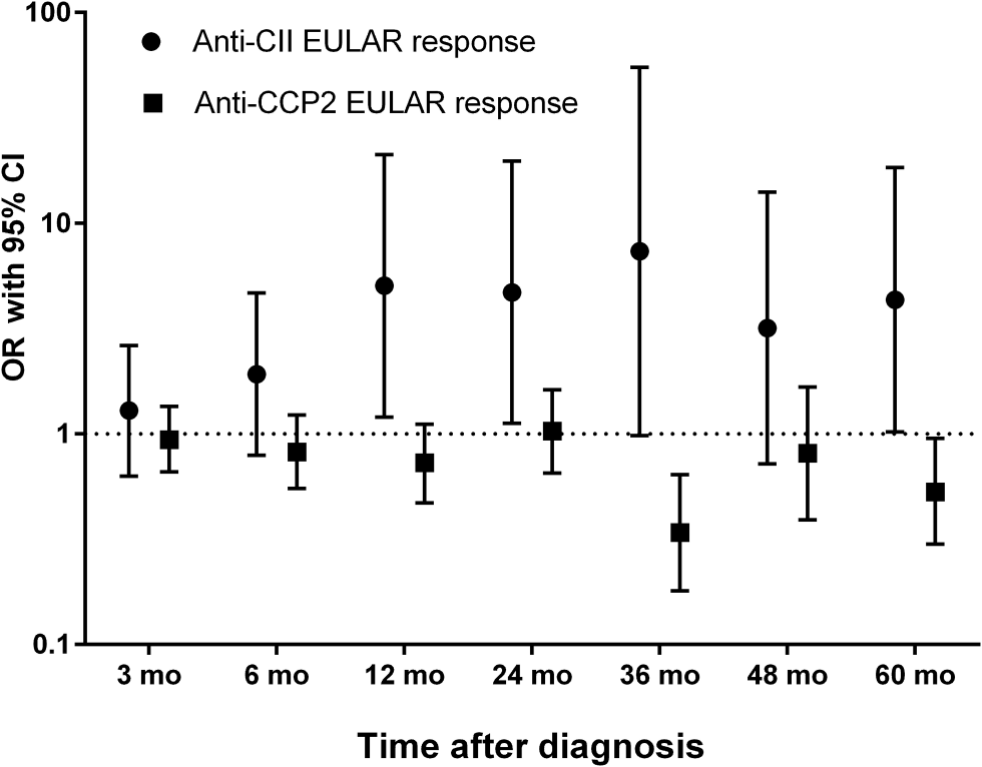
Patients with rheumatoid arthritis (RA) attaining European League Against Rheumatism(EULAR) response at the different time points, in relation to anti-CCP2 and antifibrillar collagen type II (anti-CII) autoantibody status. EULAR responses were calculated according to van Gestel *et al*, but using the EULAR recommended Disease Activity Score encompassing 28 joints (DAS28) based on erythrocyte sedimentation rate (ESR) limits as described by Jerram *et al*.^21^ ^22^ Patients achieving moderate and good EULAR response were pooled, and data are expressed as ORs with 95% CIs for attaining EULAR response. The proportion of patients receiving disease-modifying antirheumatic drug treatment at baseline did not differ between patients with and without anti-CII and anti-CCP2, respectively. Out of 773 patients, those with required clinical follow-up data were included. Full data on DAS28 components both at baseline and at the respective time point were available for 587, 559, 634, 586, 380, 229 and 435 patients at 3, 6, 12, 24, 36, 48 and 60 months, respectively. The corresponding data are shown in detail in online supplementary table S3. EULAR, European League Against Rheumatism; mo, months.

Anti-CII was associated with EULAR response at 12, 24 36 and 60 months, whereas anti-CCP2 was associated negatively to EULAR response at 36 and 60 months. Anti-CII-positive patients achieved EULAR response to a larger extent than initially DMARD-treated patients (see figure 3 and online supplementary table S5).

Anti-CII was thus associated with a favourable prognosis and anti-CCP2 with an unfavourable prognosis, compared with patients without any of the antibodies.

### Association of anti-CII and anti-CCP with HLA-DRB1* alleles

Anti-CII was positively associated with HLA-DRB1*03 (OR 1.92, 95% CI 1.23 to 2.97), and negatively with HLA-DRB1*04 (OR 0.6, 95% CI 0.39 to 0.9). After exclusion of SE-positive individuals, the HLA-DRB1*03 association remained (OR 2.82, 95% CI 1.27 to 6.28). When a higher cut-off (200 AU/mL) was employed, the positive associations with HLA-DRB1*03 (OR 3.45, 95% CI 1.73 to 6.92) and the negative association with HLA-DRB*04 (OR 0.15, 95% CI 0.06 to 0.39) were further increased, together with the appearance of positive associations with HLA-DRB1*01 (OR 2.37, 95% CI 1.18 to 4.76) and HLA-DRB1*08 (OR 2.54, 95% CI 1.02 to 6.28). After exclusion of SE-positive individuals, only the positive association with HLA-DRB1*03 remained (OR 3.4, 95% CI 1.03 to 11.25). Anti-CCP2 showed numerous HLA-DRB1* associations, all disappearing after exclusion of SE-positive patients (see online supplementary table S6).

One thousand four hundred and seventy-six patients where then divided into patients expressing only anti-CII (n=39), only anti-CCP2 (n=797) or both (n=58) and each group was individually compared with double-negative patients (n=582). Whereas patients expressing only anti-CII or anti-CCP2 showed the same HLA-DRB1* associations as described above, patients with both antibodies showed a positive association with HLA-DRB1*04 (OR=2.66, 95% CI 1.53 to 4.61) using the conventional anti-CII cut-off (see online supplementary table S7). With increased anti-CII cut-off, double antibody positivity associated with HLA-DRB1*01 (OR 3.51, 95% CI 1.25 to 9.85), whereas association with HLA-DRB1*04 was lost (not shown).

**Figure 4:**
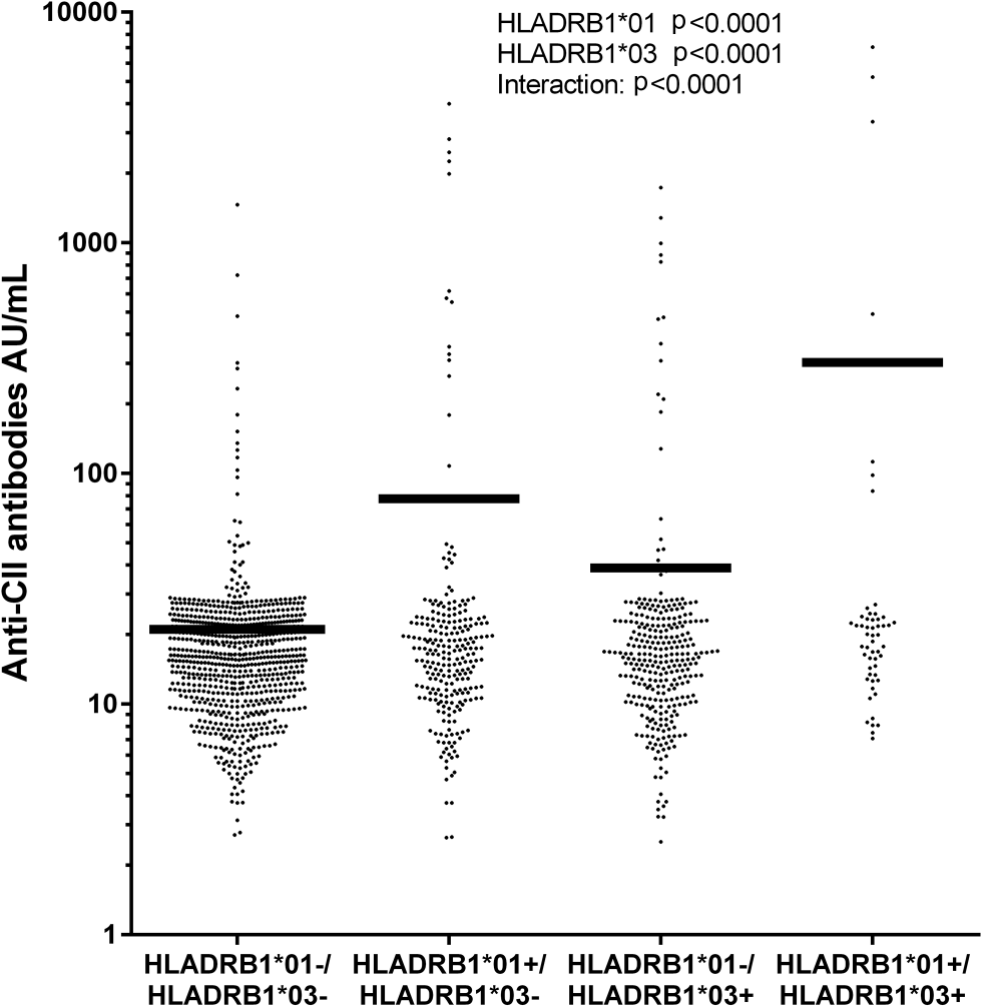
Mean levels of antibodies against native antifibrillar collagen type II (anti-CII) in relation to the occurrence of HLA-DRB1*01 and/or HLA-DRB1*03 alleles. Statistical results were obtained with two-way analysis of variance with occurrence of HLA-DRB1*01 and HLA-DRB1*03 and their interaction as independent variables and anti-CII levels as the dependent variable. Data on 103 patients with very low anti-CII levels were not depicted in the graph, but are included in the statistical calculations.

After stepwise regression with backward elimination or forward selection including HLA-DRB1*01–16, only HLA-DRB1*01 and HLA-DRB1*03 remained associated with anti-CII. Two-way ANOVA showed that both HLA-DRB1*01 and HLA-DRB1*03 were associated with anti-CII levels (p<0.0001 for both), with a highly significant interaction (p<0.0001). Whereas mean anti-CII level in HLA-DRB1*01/*03 double-negative patients (n=818) was 21.1 AU/mL, it was 77.7 AU/mL for individuals only positive for HLA-DRB1*01 (n=338) and 38.6 AU/mL for individuals only positive for HLA-DRB1*03 (n=268). The statistic interaction was manifested as strikingly increased anti-CII levels in patients with both HLA-DRB1*01 and HLA-DRB1*03 (n=52); mean 330.1 AU/mL (figure 4).

Three hundred and sixteen patients with non-specific reactivity showed no association with any HLA-DRB1* allele except a weak association with HLA-DRB1*14 (see online supplementary table S8).

### Anti-CII associated negatively with smoking

Anti-CII levels were negatively associated with smoking as ever smokers had lower anti-CII levels (median 15.2 AU/mL) compared with never smokers (median 16.5 AU/mL; p=0.0264). After exclusion of SE-positive patients, the negative association with smoking was still present (median ever smokers 14.8 AU/mL vs never smokers 17.1 AU/mL; p=0.0294). There was no difference in anti-CII levels between ever and never smoking controls (p=0.2).

Anti-CCP2 associated with smoking (median ever smokers 159 AU/mL vs never smokers 18.71 AU/mL; p=<0.0001). After exclusion of SE-positive patients, the association disappeared (7.98 vs 6.8 AU/mL; p=0.12).

## Discussion

We have shown the association of anti-CII with a distinct RA phenotype characterised by acute but transient inflammation around the time of diagnosis. Whereas we previously described increased CRP and ESR at diagnosis in anti-CII-positive patients, this acute onset phenotype has now been extended to encompass clinically relevant markers like SJC, DAS28 and EULAR response.^11^ This phenotype is in many respect the opposite to the ACPA phenotype associated with late increases in inflammatory markers and signs of disease activity; anti-CII also shows opposite associations with HLA-DRB1*03, HLA-DRB1*04 and smoking as compared with ACPA. The anti-CII-associated phenotype is strong, and predominates over the ACPA phenotype in patients with both antibodies. Most interestingly, when analysing future changes in inflammatory markers in newly diagnosed patients with RA, anti-CII is associated with a favourable outcome and anti-CCP2 with a more severe outcome both measured as changes in individual clinical and laboratory measures and attainment of EULAR response. This indicates that detection of ACPA at RA diagnosis could argue for more aggressive treatment, and the detection of anti-CII could predict a less aggressive disease course as compared with antibody-negative patients.

In a number of studies we have previously shown that anti-CII antibodies are functionally active, and that anti-CII-containing IC can stimulate MNC and PMN to cytokine and chemokine production, and probably contribute to joint erosions.^8–11^ ^20^ There is also a strong temporal association between serum anti-CII levels, in vitro function of the corresponding IC and ESR, CRP and radiological destruction.^11^ ^20^ In the previous study, as well as in the present study, antibody analyses were performed on serum samples obtained before institution of DMARD therapy. An obvious question is whether different DMARDs have divergent effects on future anti-CII levels, thus affecting anti-CII IC-driven inflammation to different degrees.

We found anti-CII in 6.6% of the patients with RA; a figure close to the 8.8% we reported previously.^11^ Although anti-CII-positive RA represent a small group as compared with ACPA, the anti-CII-associated phenotype is so profound that also the small group of patients single positive for anti-CII (2.2%; 20/773) show a strong and statistically highly significant phenotype as compared with antibody-negative patients (table 1). We believe that anti-CII measurement will contribute to the prognostic armamentarium in newly diagnosed patients with RA.

There was a positive association between anti-CII and HLA-DRB1*03 and HLA-DRB1*01, and a strong negative association with HLA-DRB1*04 and with the subtypes HLA-DRB1*0401 and HLA-DRB1*0404. The negative associations were probably second to the strong association between ACPA and SE, and disappeared after stepwise regression. Only HLA-DRB1*03 and HLA-DRB1*01 remained, and interacted, as double-positive patients had more than 14 times higher anti-CII levels than patients without HLA-DRB1*03 and HLA-DRB1*01. Three early studies on smaller groups of patients with RA (n=3160 and 166, respectively) have reported an association between HLA-DR3 and HLA-DR7 and antinative CII, but no previous studies have noted an association with HLA-DR1.^23–25^ Others have reported a negative association between ACPA and HLA-DRB1*03, although this has not been replicated in EIRA.^26^ ^27^ However, when we restrict the 797 anti-CCP2 single positive patients in online supplementary table S3 to those not expressing SE (n=116), we find a negative association with HLA-DRB1*03 (OR 0.61, 95% CI 0.38 to 0.97, data not shown), arguing that a positive association between anti-CII and HLA-DRB1*03 might mask a negative association with ACPA.

Although both the clinical phenotype and the HLA-DRB1*03 and HLA-DRB1*04 associations with anti-CII represent the counterpart to ACPA, the two antibodies were not statistically inversely related in this study as we have previously described.^11^ Anti-CII and anti-CCP2 showed very little of interaction when evaluated in two-way ANOVA against clinical measures. We interpret this that the clinical phenotypes associated with anti-CII and ACPA, respectively, exist and are regulated independent of each other.

A significant number of samples showed non-specific reactions, as, although they yielded OD levels compatible with high anti-CII levels in the ELISA, they reacted even stronger with wells that had only been blocked but did not contain the CII antigen. We believe that this reactivity is due to general ‘stickiness’, possibly related to inflammation, as we have recently described in highly inflamed *Leishmania*-infected patients.^28^ In our previous RA studies, such reactivities were treated as anti-CII-negative, but here they were treated separately. The group of non-specifically reacting patients showed none of the HLA associations found for anti-CII-positive subjects. Patients with non-specific reactivity showed early inflammatory measures in between the anti-CII-positive and anti-CII-negative patients. Although these non-specific samples showed the strongest differences when compared with the anti-CII-positive subjects, in many cases they also showed significantly higher levels as compared with anti-CII-negative patients. A plausible conclusion is that although most of these subjects lack anti-CII, they contain a group of true anti-CII-positive subjects which could not be properly identified with the ELISA. We are currently investigating alternative confirmatory techniques to extend the group of correctly identified anti-CII-positive patients.

In mice, anti-CII antibodies have been shown to be pathogenic causing acute arthritis in the non-major histocompatability complex (MHC)-dependent Collagen Antibody Induced Arthritis (CAIA) model.^29^ ^30^ We believe that early inflammation associated with anti-CII-positive RA may represent the human counterpart to CAIA. There are many similarities. We have recently shown that PMN reactivity against anti-CII IC is associated with joint destruction in RA, and that PMN+MNC cocultures stimulated with anti-CII IC produce enhanced levels of many chemokines, whereby inflammatory cells can be recruited to inflamed joints in early RA when anti-CII levels are high. The mechanism is dependent on TLR4 and functionally active granulocyte enzymes.^8^ ^10^ In rodents anti-CII induce CAIA after injection of lipopolysaccharide (LPS), a TLR4 ligand, and the ensuing polyarthritis is associated with PMN activation and can be ameliorated with a serine protease inhibitor, implying a central pathogenetic role for PMN.^31^ ^32^ The central role for TLR4 that we have described in anti-CII IC-induced production of chemokines is intriguing, as it represents an autoantibody-dependent mechanism that probably not is epitope dependent. In agreement with this, we have not been able to block anti-CII IC-stimulated chemokine production with CII peptides.

In conclusion, anti-CII-positive RA represents a distinct RA phenotype that in many ways behaves as the opposite to ACPA-associated RA concerning clinical outcome, HLA-DRB1* association and relation to smoking history. Anti-CII-positive patients with RA have an acute onset, but favourable prognosis as compared with the high disease activity at diagnosis. This opens the possibility that early detection of anti-CII together with concomitant clinical signs of elevated disease activity, might associate with a transient inflammatory phenotype, as anti-CII levels diminish during the first year and as the associated phenotype is associated with the functional activity of anti-CII, probably bound in CII-containing IC in joints.^11^ As anti-CCP2 instead is associated with poor prognosis, the combined analysis of anti-CII and ACPA/anti-CCP2 may be a new two-dimensional tool for predicting the prognosis and choosing therapy in newly diagnosed patients with RA.
