# Supplementary material for: Anticollagen type II antibodies are associated with an acute onset rheumatoid arthritis phenotype and prognosticate lower degree of inflammation during 5 years follow-up
Source: Ann Rheum Dis. 2017 Mar 23;76(9):1529–36. doi: 10.1136/annrheumdis-2016-210873 (PMC5561381; doi:10.1136/annrheumdis-2016-210873)
Supplement: supplementary data [file annrheumdis-2016-210873supp001.pdf]

Supplementary figure S1. Association between baseline elevations of anti-CII levels and a.) DAS28CRP, b.) pain VAS, c.) global VAS and d.) HAQ during five years follow-up in 773 newly diagnosed RA patients. Figures show significance levels between anti-CII positive and anti-CII negative patients at the different time points; only significant ($p<0.05$) differences and trends ($0.1<p<0.05$) are shown. Data on the same patients dichotomized according to anti-CCP status are shown in supplementary figure 2.

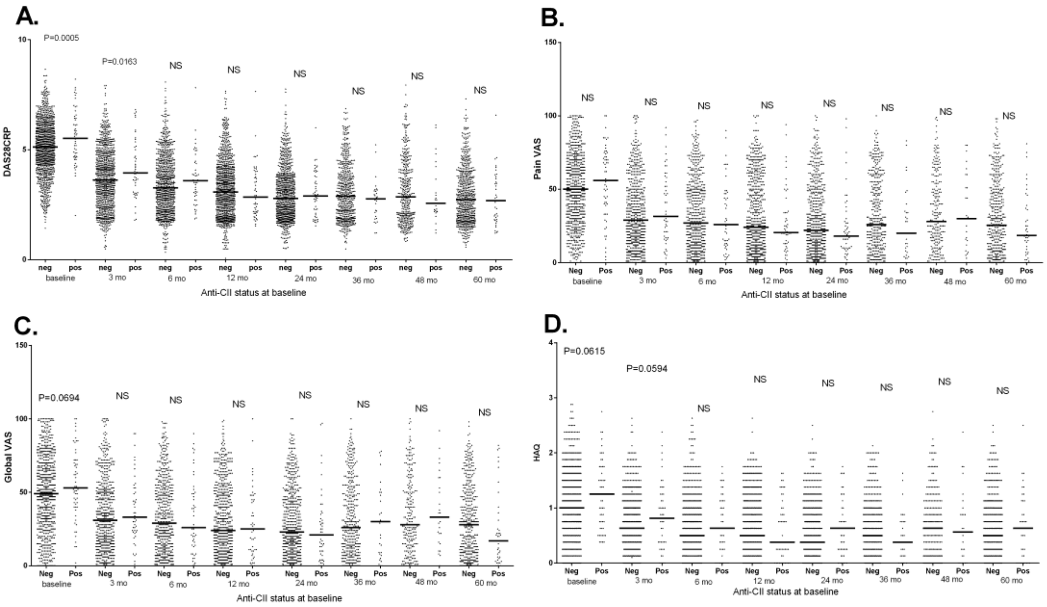

Supplementary figure S2. Association between baseline anti-CCP and a.) DAS28CRP, b.) pain VAS, c.) global VAS and d.) HAQ during five years follow-up in 773 newly diagnosed RA patients. Figures show significance levels between anti-CCP positive and anti-CCP negative patients at the different time points; only significant (p<0.05) differences and trends (0.1<p<0.05) are shown. Data on the same patients dichotomized according to anti-CII status are shown in supplementary figure 1.

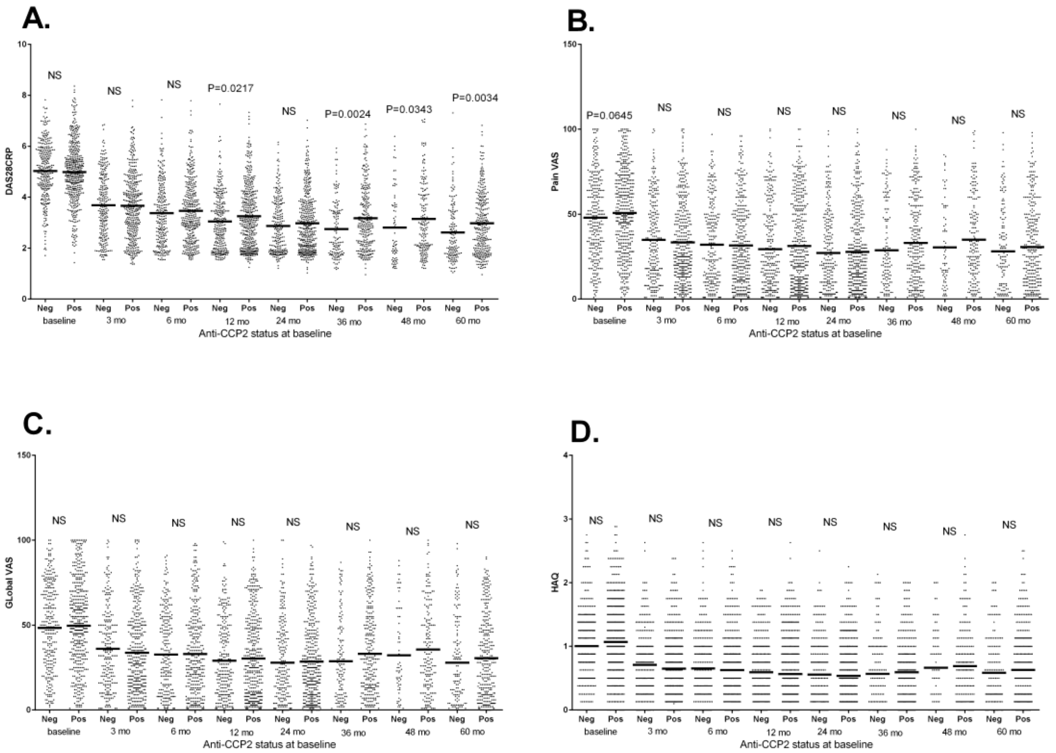

Supplementary table S1. Associations between the occurrence of non-specific anti-CII reactivity and clinical symptoms during five year follow-up after RA diagnosis. Median levels are shown for anti-CII negative subjects (n=717), anti-CII positive subjects (n=56), and patients with non-specific reactivity (n=163). Comparisons were made for laboratory and clinical variables at different time points between anti-CII negative and anti-CII positive patients, between anti-CII negative patients and patients with non-specific reactivity, and between anti-CII positive and patients with non-specific reactivity. Significant differences are depicted in bold.

Abbreviations: CRP, C reactive protein; DAS, disease activity score; ESR, erythrocyte sedimentation rate; HAQ, Health Assessment Questionnaire; VAS, Visual Analogue Scale.

	Median anti-CII negative	Median anti-CII positive	Median non-specific reactivity	P (negative vs.positive)	P (negative vs. non-specific)	P (positive vs. non-specific)
CRP 0 mo	14	31	17.5	**<0.0001**	**0.0044**	**0.0107**
CRP 3 mo	9	13	10	**0.0004**	**0.0077**	0.0796
CRP 6 mo	8	10	8	**0.0001**	0.2049	**0.0206**
CRP 12 mo	8	8	8	0.527	0.8533	0.6496
CRP 24 mo	8	8.5	6	0.2769	**0.0329**	0.7255
CRP 36 mo	7	7	8	0.5295	**0.01**	0.0612
CRP 48 mo	6	4	7	0.6957	**0.0189**	**0.0436**
CRP 60 mo	6	7	7	0.4725	0.3112	0.945
ESR 0 mo	24	40.5	29	**<0.0001**	**0.0152**	**0.0092**
ESR 3 mo	13	22	14	**0.0046**	0.2917	0.054
ESR 6 mo	12	13.5	12	0.0508	0.172	0.3485
ESR 12 mo	12	14	13	0.4097	0.7686	0.5884
ESR 24 mo	12	11	12	0.9557	0.6264	0.8033
ESR 36 mo	12	11.5	16	0.4	0.3827	0.207
ESR 48 mo	15	13	16	0.4381	0.6339	0.2943
ESR 60 mo	14	10	15	**0.0322**	0.5152	**0.0261**
SJC 0 mo	8	11.5	10	**0.0157**	0.1548	0.1125
SJC 3 mo	2	4	3	**0.0031**	0.3021	**0.0482**
SJC 6 mo	1	3	2	**0.0065**	0.2507	0.0865

SJC 12 mo	1	2	1	0.1816	0.5623	0.1491
SJC 24 mo	0	1	1	0.4859	0.0974	0.7371
SJC 36 mo	1	0	1	0.1619	0.2794	0.0766
SJC 48 mo	0	1	1	0.1945	0.1242	0.8901
SJC 60 mo	0	1	1	0.9498	0.3396	0.5153
TJC 0 mo	7	10	8	0.0229	0.4999	0.0884
TJC 3 mo	2	4	2	0.1403	0.8364	0.1701
TJC 6 mo	2	2	2	0.9727	0.6198	0.7544
TJC 12 mo	1	1	1	0.3903	0.1951	0.9594
TJC 24 mo	1	1	1	0.33	0.7652	0.4609
TJC 36 mo	1	0	1	0.1004	0.5484	0.0817
TJC 48 mo	1	0	2	0.2844	0.0683	**0.0245**
TJC 60 mo	1	1	1	0.955	0.9339	0.9468
DAS28 0 mo	5.2	5.86	5.43	**<0.0001**	0.1017	**0.0067**
DAS28 3 mo	3.53	3.98	3.5	**0.0057**	0.8031	**0.0297**
DAS28 6 mo	3.24	3.51	3.24	0.1629	0.6968	0.1685
DAS28 12 mo	2.99	2.95	2.49	0.7424	0.0924	0.5664
DAS28 24 mo	2.8	2.9	2.78	0.6924	0.8405	0.8997
DAS28 36 mo	2.93	2.79	3	0.1584	0.5887	0.141
DAS28 48 mo	2.97	2.86	3.44	0.7307	0.3413	0.3924
DAS28 60 mo	2.85	2.45	2.65	0.1542	0.8436	0.248
DAS28CRP 0 mo	4.96	5.52	5.15	**0.0005**	0.2171	**0.0156**
DAS28CRP 3 mo	3.56	3.95	3.59	**0.0163**	0.6405	0.0788
DAS28CRP 6 mo	3.23	3.59	3.25	0.0993	0.8595	0.1675
DAS28CRP 12 mo	3.06	2.84	2.72	0.6647	0.0521	0.4497
DAS28CRP 24 mo	2.72	2.9	2.66	0.5609	0.5886	0.7856
DAS28CRP 36 mo	2.87	2.76	2.86	0.1817	0.2819	0.1249
DAS28CRP 48 mo	2.8	2.56	3.16	0.808	0.0742	0.3513
DAS28CRP 60 mo	2.64	2.67	2.58	0.7133	0.8677	0.8076
Pain 0 mo	50	56	50	0.1274	0.2952	0.4138
Pain 3 mo	29	31.5	28	0.2246	0.9379	0.2948
Pain 6 mo	27	26	27	0.9683	0.7479	0.7934
Pain 12 mo	24	20.5	18	0.6331	0.0911	0.5059
Pain 24 mo	22	18	22	0.4527	0.8435	0.4617
Pain 36 mo	26	20	26	0.2703	0.697	0.4243
Pain 48 mo	28	30	27	0.7994	0.7498	0.6476
Pain 60 mo	25.5	18.5	25	0.2847	0.815	0.2317
Global VAS 0 mo	49	53	50	0.0694	0.9155	0.0997
Global VAS 3 mo	31	33	29	0.2229	0.8307	0.2669
Global VAS 6 mo	29	26	24	0.7072	0.1287	0.1879
Global VAS 12 mo	24	25	17	0.9241	**0.0413**	0.2653

Global VAS 24 mo	23	21	23	0.333	0.8077	0.4932
Global VAS 36 mo	26	30	32	0.8118	0.4971	0.4885
Global VAS 48 mo	28	33	28	0.5041	0.3114	0.2088
Global VAS 60 mo	28	17	20	0.1138	0.3121	0.2838
HAQ 0 mo	1	1.25	1	0.0615	0.767	0.128
HAQ 3 mo	0.63	0.82	0.63	0.0594	0.9172	0.097
HAQ 6 mo	0.5	0.63	0.5	0.5863	0.8941	0.59
HAQ 12 mo	0.5	0.38	0.38	0.6822	0.5167	0.4699
HAQ 24 mo	0.38	0.63	0.5	0.1399	0.7764	0.328
HAQ 36 mo	0.5	0.38	0.57	0.4872	0.2652	0.2183
HAQ 48 mo	0.63	0.57	0.75	0.7792	1	0.9002
HAQ 60 mo	0.5	0.63	0.5	0.6186	0.9421	0.6748

Supplementary Table S2. Associations between the occurrence of anti-CII and anti-CCP, individually or in combination and clinical symptoms during five year follow-up after RA diagnosis. Median levels are shown for anti-CII/anti-CCP double negative patients (n=285), anti-CII positive and anti-CCP negative patients (n=20), anti-CII negative and anti-CCP positive patients (n=432) and anti-CII positive and anti-CCP2 positive patients (n=36). P values refer to comparisons with the anti-CII negative/anti-CCP2 double negative group. Significant differences are depicted in bold, and also underlined if the median level for the corresponding antibody is lower than for the double negative group. Abbreviations: TJC, Tender joint count; CRP, C-reactive protein; DAS, disease activity score; HAQ, Health Assessment Questionnaire; RA, rheumatoid arthritis; VAS, Visual Analogue Scale. This table is an amendment to Table 1, including clinical variables not shown in the printed paper.

Variable	Median anti-CII-/ anti-CCP-	Median anti-CCP+	Median anti-CII+	Median anti-CII+/ anti-CCP+	P (Anti-CCP)	P (Anti-CII)	P (Anti-CII+/ Anti-CCP+)
TJC 0 mo	8	7	9	10	0.0507	0.3287	0.2184
TJC 3 mo	2	2	4	3	0.2769	0.198	0.8287
TJC 6 mo	2	2	1.5	2	0.8609	0.6959	0.6709
TJC 12 mo	1	1	1	1	0.7637	0.9806	0.3739
TJC 24 mo	1	1	1	1	0.3927	0.6795	0.3084
TJC 36 mo	0	1	0	1	**0.0387**	0.4851	0.688
TJC 48 mo	1	1	0	1	0.7175	0.2582	0.877
TJC 60 mo	0	1	0.5	1	0.1168	0.955	0.4795
DAS28CRP 0 mo	5.01	4.93	5.69	5.3	0.4599	**0.0126**	**0.0273**
DAS28CRP 3 mo	3.51	3.59	3.96	3.7	0.8824	0.045	0.1725
DAS28CRP 6 mo	3.24	3.22	3.29	3.8	0.5429	0.9256	0.0295
DAS28CRP 12 mo	2.9	3.09	2.44	3.13	0.0655	0.4961	0.4899
DAS28CRP 24 mo	2.69	2.75	2.58	2.96	0.455	0.6495	0.1766
DAS28CRP 36 mo	2.39	2.96	2.05	2.85	**0.0014**	0.1715	0.4875
DAS28CRP 48 mo	2.16	2.91	2.27	2.71	**0.0422**	1	0.154
DAS28CRP 60 mo	2.27	2.79	2.01	2.99	**0.0043**	0.4504	0.1094
Pain 0 mo	47.44	50	62	53.36	0.0867	0.1046	0.1817
Pain 3 mo	33	27	25	41	0.2691	0.5751	0.1586
Pain 6 mo	28	26	15	30	0.6063	0.1525	0.3272
Pain 12 mo	22	25	20	22	0.5826	0.2837	0.6377
Pain 24 mo	21	22	16	20	0.7724	0.3119	0.9282
Pain 36 mo	22	28	8	30	0.1455	0.0788	0.4749
Pain 48 mo	22.5	28	18.5	32	0.3228	0.5129	0.2373
Pain 60 mo	25	26	12	21.5	0.4503	0.0967	0.7511

Global VAS 0 mo	48	49	51	54	0.6439	0.2666	0.1033
Global VAS 3 mo	34	29	32	34	0.1584	0.8349	0.5082
Global VAS 6 mo	31	28	11	39	0.4504	0.0537	0.1054
Global VAS 12 mo	23	25	19	29	0.9185	0.274	0.48
Global VAS 24 mo	23	23.5	19.5	21	0.6797	0.4559	0.6686
Global VAS 36 mo	24	27	8	33	0.191	0.1673	0.188
Global VAS 48 mo	25	29	24.5	36	0.293	0.771	0.263
Global VAS 60 mo	21	28	11.5	20	0.2674	0.1292	0.9713
HAQ 0 mo	1	1	1.5	1	0.157	**0.0095**	0.2929
HAQ 3 mo	0.63	0.5	0.75	0.88	0.1198	0.4464	0.2667
HAQ 6 mo	0.63	0.5	0.44	0.75	0.2327	0.2373	0.2479
HAQ 12 mo	0.5	0.38	0.38	0.44	0.2196	0.5809	0.6573
HAQ 24 mo	0.38	0.38	0.38	0.63	0.6374	0.8408	0.1708
HAQ 36 mo	0.5	0.5	0.13	0.69	0.8679	0.0603	0.5447
HAQ 48 mo	0.63	0.63	0.315	0.755	0.9281	0.2957	0.7487
HAQ 60 mo	0.38	0.5	0.375	0.75	0.9722	0.4961	0.2562

Supplementary table S3. Association between inflammatory markers and the occurrence of anti-CII and anti-CCP2 at the time of RA diagnosis. Analysis was performed with two-way ANOVA. Mean levels are shown for anti-CII/anti-CCP double negative subjects (n=285), anti-CII positive and anti-CCP negative subjects (n=20), anti-CII negative and anti-CCP positive patients (n=432) and anti-CII positive anti-CCP2 positive patients n=36). P values for the total ANOVA, anti-CCP2, anti-CII and the interaction between anti-CII and anti-CCP2 are given in individual columns. Significant p values are depicted in bold.

Symptoms	Mean anti-CCP-/anti-CII-	Mean anti-CCP-/anti-CII+	Mean Anti-CCP+/ anti-CII-	Mean Anti-CCP+/ anti-CII+	ANOVA Total p	Anti-CCP p	Anti-CII p	interaction
CRP 0 mo	21.76	42.5	29.24	43.2	**<0.0001**	0.3964	**0.0003**	0.482
CRP 3 mo	12.59	19.82	16.29	24.62	**0.0047**	0.166	**0.0113**	0.8573
CRP 6 mo	11.76	16.56	14.73	22.77	**0.0211**	0.1321	**0.0354**	0.5949
CRP 12 mo	11.58	11.39	14.67	16.79	0.2106	0.1778	0.7592	0.7128
CRP 24 mo	8.54	8.44	10.91	11.31	**0.0273**	0.0828	0.9199	0.8695
CRP 36 mo	9.24	4.5	11.93	15.95	0.084	0.0205	0.9054	0.1499
CRP 48 mo	7.58	10	12.65	11	0.2679	0.4448	0.9223	0.6082
CRP 60 mo	7.94	4.07	8.96	15.27	**0.0265**	**0.006**	0.5814	**0.0218**
ESR 0 mo	25.56	37.21	33.05	49.88	**<0.0001**	**0.0028**	**<0.0001**	0.4416
ESR 3 mo	16.38	15.73	21.41	32.46	**<0.0001**	**0.0003**	0.0799	**0.0491**
ESR 6 mo	1.13	4.05	0.94	3.1	**0.0005**	**0.0007**	0.1265	**0.0262**
ESR 12 mo	1.02	3.89	0.83	2.84	**0.0001**	**0.0002**	0.4857	**0.0387**
ESR 24 mo	1.02	3.57	0.76	2.6	0.05	0.0126	0.846	0.1438
ESR 36 mo	1.45	4.96	1.03	3.78	**0.0042**	**0.0095**	0.3661	0.3663
ESR 48 mo	16.89	12.43	21.81	18.61	0.157	0.1767	0.3507	0.8772
ESR 60 mo	15.77	<u>7.93</u>	19.5	19.16	**0.0036**	**0.0027**	0.1	0.1312
SJC 0 mo	9.58	13.6	9.06	11	**0.0016**	0.0584	**0.0003**	0.208
SJC 3 mo	3.76	6.76	3.95	6.31	**0.0026**	0.8539	**0.0002**	0.6542
SJC 6 mo	2.54	5.5	3.14	5.4	**0.0002**	0.6971	**<0.0001**	0.586
SJC 12 mo	1.68	3.26	2.67	3.59	**0.0005**	0.2122	**0.0183**	0.5279
SJC 24 mo	1.4	0.94	2.02	2	0.061	0.0768	0.6183	0.6489
SJC 36 mo	1.27	1	2.67	1.2	**0.0002**	0.1936	0.1579	0.3295
SJC 48 mo	1.82	3.13	2.48	3.95	0.1625	0.3766	0.0986	0.9221
SJC 60 mo	1.5	<u>0.64</u>	2.3	2.46	**0.0499**	**0.0284**	0.5615	0.396
TJC 0 mo	9.17	10.7	7.96	10.5	**0.0066**	0.4369	0.0257	0.577
TJC 3 mo	4.79	5.59	3.78	4.86	**0.0598**	0.2829	0.2453	0.8596
TJC 6 mo	3.83	4.33	3.37	3	0.5657	0.2409	0.9285	0.5651
TJC 12 mo	2.92	3.63	2.91	1.68	0.3473	0.1237	0.681	0.1269
TJC 24 mo	2.52	1.72	2.19	2.32	0.6159	0.8065	0.5371	0.388

TJC 36 mo	2.15	1.27	2.71	1.05	0.095	0.8096	0.0737	0.5783
TJC 48 mo	2.97	2.13	2.81	2.05	0.8504	0.9063	0.4242	0.9638
TJC 60 mo	1.91	1.29	2.19	1.85	0.6291	0.4501	0.3819	0.8001
DAS28 0 mo	5.09	5.93	5.17	6.05	**<0.0001**	0.6038	**<0.0001**	0.9121
DAS28 3 mo	3.59	4.08	3.65	4.47	**0.0173**	0.3416	**0.0074**	0.488
DAS28 6 mo	3.27	3.27	3.39	3.91	0.1683	0.1001	0.2678	0.2647
DAS28 12 mo	2.94	2.8	3.22	3.19	0.0909	0.1397	0.7019	0.8089
DAS28 24 mo	2.84	2.56	2.99	3.19	0.2438	0.0605	0.8562	0.2404
DAS28 36 mo	2.77	2.28	3.32	2.94	**0.0006**	**0.0264**	0.1062	0.8442
DAS28 48 mo	2.99	2.84	3.37	3.25	0.3784	0.2904	0.7086	0.9702
DAS28 60 mo	2.72	2.21	3.17	3.01	**0.0018**	**0.0082**	0.1559	0.4718
DAS28CRP 0 mo	4.98	5.66	4.93	5.57	**0.0012**	0.7021	**0.0001**	0.9101
DAS28CRP 3 mo	3.64	4.27	3.63	4.1	0.0559	0.6357	0.0065	0.6776
DAS28CRP 6 mo	3.36	3.48	3.43	3.81	0.3246	0.3039	0.1937	0.4998
DAS28CRP 12 mo	3.04	3.08	3.26	3.1	0.1435	0.5104	0.7354	0.5998
DAS28CRP 24 mo	2.88	2.73	2.96	3.13	0.5218	0.1624	0.9734	0.3461
DAS28CRP 36 mo	2.78	2.3	3.19	2.9	**0.0031**	**0.0387**	0.1108	0.7061
DAS28CRP 48 mo	2.81	2.78	3.15	3.12	0.4068	0.2894	0.9239	0.9946
DAS28CRP 60 mo	2.65	2.3	2.97	3.05	**0.0171**	**0.0103**	0.5072	0.2962
Pain 0 mo	47.44	55.4	50.54	53.36	0.1953	0.8822	0.1297	0.4706
Pain 3 mo	35.23	30.47	32.74	42.24	0.1435	0.2218	0.5323	0.061
Pain 6 mo	32.68	22.72	31.2	36.69	0.2476	0.1024	0.5587	0.0434
Pain 12 mo	29.99	21.11	31.31	30.74	0.3905	0.1607	0.2263	0.2865
Pain 24 mo	27.66	21.05	27.99	25.87	0.6595	0.4869	0.2389	0.545
Pain 36 mo	29.56	18.63	33.33	30.52	0.1701	0.1154	0.1682	0.415
Pain 48 mo	31.13	25.13	34.51	38.74	0.4702	0.1314	0.8747	0.3632
Pain 60 mo	29.44	15.93	30.65	30.35	0.1897	0.0698	0.1089	0.1255
Global VAS 0 mo	47.9	54.9	49.07	55.89	0.2677	0.777	0.0697	0.9814
Global VAS 3 mo	36.14	35.53	33.35	39.48	0.3833	0.8817	0.4783	0.3862
Global VAS 6 mo	33.47	21.94	32.31	41.55	0.0688	0.0191	0.7707	0.0084
Global VAS 12 mo	29.41	23.05	30.15	32.16	0.6016	0.1853	0.5579	0.2596
Global VAS 24 mo	28.28	23	28.63	26.16	0.7489	0.6318	0.2901	0.701
Global VAS 36 mo	29.32	21.36	32.93	35.05	0.2695	0.0815	0.5556	0.3095
Global VAS 48 mo	31.84	35.13	35.34	38.44	0.7344	0.5544	0.5793	0.9878
Global VAS 60 mo	28.91	17.86	30.79	27.63	0.2525	0.1739	0.0973	0.3565
HAQ 0 mo	0.98	1.32	1.06	1.15	**0.0474**	0.5871	**0.0178**	0.1599
HAQ 3 mo	0.71	0.77	0.63	0.88	0.0579	0.838	0.0749	0.2894
HAQ 6 mo	0.67	0.48	0.61	0.78	0.2149	0.1572	0.9236	0.0446
HAQ 12 mo	0.6	0.51	0.56	0.64	0.6745	0.6052	0.9944	0.3145
HAQ 24 mo	0.56	0.54	0.53	0.68	0.4763	0.5197	0.4267	0.307
HAQ 36 mo	0.59	0.26	0.59	0.63	0.2612	0.0952	0.1723	0.0938
HAQ 48 mo	0.69	0.49	0.68	0.76	0.7611	0.3194	0.6516	0.3006
HAQ 60 mo	0.6	0.48	0.62	0.76	0.4615	0.1299	0.9106	0.1942

Supplementary Table S4. Association between changes in inflammatory markers as compared to baseline values and the occurrence of anti-CII and anti-CCP2 at the time of RA diagnosis. Analysis was performed with two-way ANOVA, and changes in clinical and laboratory measures were expressed as differences between values at different time points and corresponding baseline values. Mean levels are shown for anti-CII/anti-CCP double negative subjects (n=285), anti-CII positive and anti-CCP negative subjects (n=20), anti-CII negative and anti-CCP positive patients (n=432) and anti-CII positive anti-CCP2 positive patients n=36). P values for the total ANOVA, anti-CCP2, anti-CII and the interaction between anti-CII and anti-CCP2 are given in individual columns. Significant p values for the individual antibodies are depicted in bold, and also underlined if the mean level for the corresponding antibody is lower than for the double negative group. Abbreviations: TJC, Tender joint count; CRP, C-reactive protein; DAS, disease activity score; HAQ, Health Assessment Questionnaire; RA, rheumatoid arthritis; VAS, Visual Analogue Scale. This table is an amendment to Table 2, including clinical variables not shown in the printed paper.

Symptom changes	Mean CCP- CII-	Mean CCP+ CII-	Mean CCP- CII+	Mean CCP+ CII+	ANOVA Total p	Anti-CCP p	Anti-CII p	interaction
TJC Δ 3 mo	-4.6	-4.14	-4.76	-5	0.7911	0.9162	0.6259	0.7427
TJC Δ 6 mo	-5.4	-4.53	-6.55	-8.2	**0.0161**	0.7055	**0.0194**	0.2228
TJC Δ 12 mo	-6.48	-4.97	-7.63	-8.5	**0.0008**	0.7362	**0.0156**	0.2167
TJC Δ 24 mo	-6.94	-5.8	-9.94	-7.94	**0.0048**	0.1006	**0.0075**	0.6514
TJC Δ 36 mo	-7.85	-5.61	-12.36	-9.2	**0.0002**	**0.0463**	**0.0029**	0.7326
TJC Δ 48 mo	-7.27	-6.11	-10.25	-9.68	0.0843	0.5912	0.0424	0.8531
TJC Δ 60 mo	-6.97	-5.8	-9.79	-8.81	**0.0157**	0.3534	**0.0121**	0.9344
DAS28CRP Δ 3 mo	-1.37	-1.29	-1.47	-1.37	0.8864	0.6873	0.6741	0.9525
DAS28CRP Δ 6 mo	-1.62	-1.53	-2.33	-1.89	0.0718	0.2244	0.0152	0.4341
DAS28CRP Δ 12 mo	-1.97	-1.68	-2.83	-2.25	**0.0005**	**0.0499**	**0.0014**	0.5187
DAS28CRP Δ 24 mo	-2.15	-1.98	-3.2	-2.37	**0.0038**	**0.0337**	**0.0017**	0.1733
DAS28CRP Δ 36 mo	-2.32	-1.81	-3.74	-2.6	**<0.0001**	**0.0085**	**0.0005**	0.3152
DAS28CRP Δ 48 mo	-2.4	-2.05	-3.09	-2.84	0.1116	0.4615	0.0647	0.9052
DAS28CRP Δ 60 mo	-2.39	-2.03	-3.56	-2.56	**0.0013**	0.0164	**0.0029**	0.261
Pain Δ 3 mo	-12.38	-17.82	-27.94	-8.03	**0.0168**	0.1115	0.5251	**0.0054**
Pain Δ 6 mo	-15.68	-19.22	-34.72	-16.31	**0.0481**	0.1049	0.0787	**0.0168**
Pain Δ 12 mo	-17.68	-19.14	-37.11	-20.74	0.0547	0.0973	0.0195	0.0476
Pain Δ 24 mo	-20.96	-21.92	-38.56	-28.06	0.0736	0.3009	0.0102	0.2144

Pain Δ 36 mo	-22.2	-17.49	-41.45	-26.05	**0.0432**	0.1029	**0.0244**	0.3859
Pain Δ 48 mo	-22.29	-20.65	-31.13	-29.37	0.575	0.8104	0.2163	0.9937
Pain Δ 60 mo	-20.37	-20.9	-44.71	-25.69	**0.0386**	0.0893	**0.0076**	0.0724
Global VAS Δ 3 mo	-11.67	-15.35	-22.12	-12.79	0.3303	0.5528	0.4066	0.1712
Global VAS Δ 6 mo	-15.36	-16.61	-34.89	-15.31	0.0707	0.0519	0.053	0.0271
Global VAS Δ 12 mo	-18.64	-18.58	-34.05	-21.23	0.1894	0.1673	0.053	0.1708
Global VAS Δ 24 mo	-19.7	-20.38	-33.94	-29.87	0.1017	0.7216	0.0131	0.6188
Global VAS Δ 36 mo	-20.15	-17.32	-39.45	-24.65	0.1227	0.1653	0.0364	0.3458
Global VAS Δ 48 mo	-23.69	-18.83	-21.75	-25.89	0.7036	0.9624	0.7363	0.5541
Global VAS Δ 60 mo	-21.65	-18.44	-41.29	-32.33	**0.0138**	0.2759	**0.0028**	0.6071
HAQ Δ 3 mo	-0.29	-0.44	-0.57	-0.25	**0.0048**	0.3133	0.6265	**0.0085**
HAQ Δ 6 mo	-0.33	-0.44	-0.85	-0.43	**0.0015**	0.1178	**0.0068**	**0.0041**
HAQ Δ 12 mo	-0.38	-0.49	-0.83	-0.5	**0.0055**	0.2049	**0.0097**	**0.0153**
HAQ Δ 24 mo	-0.42	-0.52	-0.85	-0.47	**0.023**	0.1448	**0.0414**	**0.0135**
HAQ Δ 36 mo	-0.43	-0.48	-1.24	-0.64	**0.0004**	**0.0266**	**<0.0001**	**0.0074**
HAQ Δ 48 mo	-0.37	-0.57	-0.93	-0.63	0.0797	0.7672	0.0532	0.1139
HAQ Δ 60 mo	-0.4	-0.48	-0.93	-0.4	**0.0431**	0.0534	0.054	**0.0092**

Supplementary table S5. RA patients attaining EULAR response at the different time points, in relation to anti-CCP2 and anti-CII autoantibody status. EULAR responses were calculated according to van Gestel et al (Arthritis Rheum 1996;39:34), but using the EULAR recommended DAS28 limits as described by Jerram et al (Rheumatology 2008;47:180), and patients achieving moderate and good EULAR response were pooled. Out of 773 patients, those with required clinical follow-up data were included. Full data on DAS28 components both at baseline and at the respective time point were available for 587, 559, 634, 586, 380, 229 and 435 patients at 3, 6, 12, 24, 36, 48 and 60 months, respectively. Significant differences and OR values are depicted in bold. The corresponding data are graphically shown in figure 3.

	Anti-CCP + Responders /non responders	Anti-CCP - Responders /non responders	OR (CI_{low}-CI_{high})	p	Anti-CII + Responders /non responders	Anti-CII - Responders /non responders	OR (CI_{low}-CI_{high})	p
EULAR response 3 months	248/114	157/68	0.94(0.66-1.35)	0.7465	31/11	374/171	1.29 (0.63-2.62)	0.4838
EULAR response 6 months	251/79	182/47	0.82(0.55-1.23)	0.3419	38/6	395/120	1.92 (0.79-4.66)	0.1409
EULAR response 12 months	308/76	212/38	0.73(0.47-1.11)	0.1412	43/2	477/112	**5.05 (1.2-21.15)**	**0.0142**
EULAR response 24 months	313/60	178/35	1.03(0.65-1.62)	0.9129	45/2	446/93	**4.69 (1.12-19.68)**	**0.0204**
EULAR response 36 months	188/61	118/13	**0.34(0.18-0.64)**	**0.0006**	28/1	278/73	7.35 (0.98-54.94)	**0.0234**
EULAR response 48 months	128/36	53/12	0.81(0.39-1.67)	0.5586	22/2	159/46	3.18 (0.72-14.03)	0.1082
EULAR response 60 months	235/62	121/17	**0.53(0.3-0.95)**	**0.0312**	36/2	320/77	**4.33 (1.02-18.38)**	**0.0309**

Supplementary Table S6. Odds ratios (OR) and 95% confidence intervals (CI) for the occurrence of anti-CII or anti-CCP in relation to individual HLA-DRB1* alleles in 1476 RA patients. Anti-CII positivity has been defined according to two different cutoffs: 29 AU/ml, representing the 95th percentile of healthy controls (Mullazehi M et al, Ann Rheum Dis 2007;66;537), and 200 AU/ml representing the value corresponding to cytokine induction from PBMC by anti-CII-containing surface-bound immune complexes *in vitro*. (Mullazehi M et al, Arthritis Rheum 2006;54:1759 and Mullazehi M et al. Arthritis Res Ther 2012;14(3):R100) Data are shown both for all patients as well as excluding patients expressing any of the HLA-DRB1* shared epitope alleles, as the strong association between ACPA and shared epitope skewed the HLA associations noted for anti-CII.

**Anti-CII >29AU/ml**		**All patients (n=1476)**				**SE negative patients (n=400)**		
	Anti-CII pos with/without genotype	Anti-CII neg with/without genotype	OR(CI_{low}-CI_{high})	p value	Anti-CII pos with/without genotype	Anti-CII neg with/without genotype	OR(CI_{low}-CI_{high})	p value
**DRB1*01**	30/67	360/1019	1.27(0.81-1.98)	0.2978	0/28	4/368	0	0.5813
**DRB1*03**	33/64	287/1092	**1.96(1.26-3.05)**	**0.0023**	18/10	141/231	**2.95 (1.32-6.57)**	**0.0059**
**DRB1*04**	40/57	746/633	**0.6(0.39-0.9)**	**0.0141**	0/28	9/363	0	0.4051
**DRB1*07**	9/88	132/1247	0.96(0.47-1.96)	0.9242	5/23	72/300	0.91(0.33-2.46)	0.8463
**DRB1*08**	11/86	111/1268	1.46(0.75-2.81)	0.2552	6/22	54/318	1.61(0.62-4.14)	0.3232
**DRB1*09**	3/94	52/1327	0.81(0.24-2.65)	0.7333	1/27	24/348	0.54(0.07-4.12)	0.5437
**DRB1*10**	3/94	49/1330	0.86(0.27-2.83)	0.6843				
**DRB1*11**	4/93	114/1265	0.48(0.17-1.32)	0.1459	2/26	59/313	0.41(0.09-1.77)	0.2159
**DRB1*12**	5/92	51/1328	1.41(0.55-3.63)	0.4681	1/27	23/349	0.56(0.07-4.32)	0.5747
**DRB1*13**	19/78	227/1152	1.23(0.73-2.08)	0.4245	9/19	125/247	0.94(0.412-2.13)	0.8746
**DRB1*14**	2/95	41/1338	0.68(0.16-2.88)	0.606	1/27	25/347	0.51(0.07-3.94)	0.5145
**DRB1*15**	18/79	320/1059	0.75(0.44-1.28)	0.2923	8/20	137/235	0.69(0.29-1.6)	0.3808
**DRB1*16**	1/96	22/1357	0.64(0.09-4.81)	0.6644	1/27	14/358	0.95(0.12-7.48)	0.9589
**DRB04*01**	22/75	452/927	**0.6(0.36-0.98)**	**0.0395**				
**DRB04*04**	12/85	155/1224	1.11(0.6-2.09)	0.7339				
**SE**	69/28	1007/372	0.91(0.58-1.43)	0.6856				

Anti-CII >200AU/ml		All patients (n=1476)				SE negative patients (n=400)		
	Anti-CII pos with/without genotype	Anti-CII neg with/without genotype	OR(CI_{low}-CI_{high})	p value	Anti-CII pos with/without genotype	Anti-CII neg with/without genotype	OR(CI_{low}-CI_{high})	p value
**DRB1*01**	15/18	375/1068	**2.37(1.18-4.76)**	**0.0121**	0/13	4/383	0	0.7126
**DRB1*03**	16/17	304/1139	**3.53(1.76-7.06)**	**0.0002**	9/4	150/237	**3.56(1.08-11.75)**	**0.0272**
**DRB1*04**	5/28	781/662	**0.15(0.06-0.39)**	**<0.0001**	0/13	9/378	0	0.5781
**DRB1*07**	5/28	136/1307	1.71(0.65-4.51)	0.2685	3/10	74/313	1.27(0.34-4.73)	0.722
**DRB1*08**	6/27	116/1327	**2.54(1.02-6.28)**	**0.0364**	4/9	56/331	2.63(0.78-8.82)	0.1055
**DRB1*09**	0/33	52/1391	0	0.2669	0/13	25/362	0	0.3439
**DRB1*10**	0/33	57/1386	0	0.2442				
**DRB1*11**	1/32	117/1326	0.35(0.05-2.62)	0.2876	1/12	60/327	0.45(0.06-3.56)	0.4409
**DRB1*12**	1/32	55/1388	0.79(0.11-5.88)	0.8163	1/12	23/364	1.32(0.16-10.59)	0.7939
**DRB1*13**	8/25	238/1205	1.62(0.72-3.64)	0.2376	4/9	130/257	0.89(0.27-2.91)	0.832
**DRB1*14**	2/31	41/1402	2.2(0.51-9.53)	0.2769	1/12	25/362	1.21(0.15-9.66)	0.8593
**DRB1*15**	5/28	333/1110	0.6(0.23-1.55)	0.284	2/11	143/244	0.31(0.07-1.42)	0.1116
**DRB1*16**	0/33	23/1420	0	0.4648	0/13	15/372	0	0.4693
**DRB04*01**	2/31	472/971	**0.13(0.03-0.56)**	**0.0012**				
**DRB04*04**	1/32	166/1277	0.24(0.03-1.78)	0.1287				
**SE**	20/13	1056/387	0.56(0.28-1.14)	0.1081				

Anti-CCP		All patients (n=1476)				SE negative patients (n=400)		
	Anti-CCP2 pos with/without genotype	Anti-CCP2 neg with/without genotype	OR(CI_{low}-CI_{high})	p value	Anti-CCP2 pos with/without genotype	Anti-CCP2 neg with/without genotype	OR(CI_{low}-CI_{high})	p value
**DRB1*01**	235/620	155/466	1.14(0.9-1.44)	0.2773	1/124	3/172	0.73(0.08-7.1)	0.7821
**DRB1*03**	138/717	182/439	**0.46(0.36-0.6)**	**<0.0001**	41/84	118/157	0.65(0.42-1.01)	0.0555
**DRB1*04**	569/286	217/404	**3.7(2.97-4.6)**	**<0.0001**	3/122	6/269	1.1(0.27-4.48)	0.8915
**DRB1*07**	75/780	66/555	0.81(0.57-1.15)	0.2311	31/94	46/229	1.64(0.98-2.75)	0.058
**DRB1*08**	41/814	81/540	**0.34(0.23-0.5)**	**<0.0001**	12/113	48/227	**0.5(0.26-0.98)**	**0.0414**
**DRB1*09**	33/822	22/599	1.09(0.63-1.89)	0.7509	11/114	14/261	1.8(0.79-4.08)	0.1555
**DRB1*10**	39/816	13/608	**2.24(1.18-4.22)**	**0.0111**				
**DRB1*11**	57/798	61/560	**0.66(0.45-0.96)**	**0.0273**	21/104	40/235	1.19(0.67-2.11)	0.561
**DRB1*12**	29/826	27/594	0.77(0.45-1.32)	0.3426	6/119	18/257	0.72(0.28-1.86)	0.4957
**DRB1*13**	85/770	161/460	**0.32(0.24-0.42)**	**<0.0001**	35/90	99/176	0.69(0.44-1.1)	0.1161

**DRB1*14**	18/837	25/596	**0.51(0.28-0.95)**	**0.0303**	7/118	19/256	0.8(0.33-1.96)	0.6225
**DRB1*15**	193/662	145/476	0.96(0.75-1.22)	0.726	56/69	89/186	1.7(1.1-2.62)	0.0165
**DRB1*16**	14/841	9/612	1.13(0.49-2.63)	0.7732	8/117	7/268	2.62(0.93-7.39)	0.06
**DRB04*01**	352/503	122/499	**2.86(2.25-3.64)**	**<0.0001**				
**DRB04*04**	120/735	47/574	**1.99(1.4-2.84)**	**0.0001**				
**SE**	730/125	346/275	**4.64(3.62-5.94)**	**<0.0001**				

Supplementary table S7. Odds ratios (OR) and 95% confidence intervals (CI) for 1476 RA patients evaluated separately for patients with only anti-CCP2 reactivity (n=797), only anti-CII reactivity (n=39), or anti-CII + anti-CCP2 double reactivity (n=58) in relation to individual HLA-DRB1* alleles in RA patients. In each comparison the antibody positive patients were compared to patients negative for both anti-CII and anti-CCP (n=582). Significant associations are depicted in bold.

Anti-CCP single positive	Anti-CCP2 positive with/without genotype	Anti-CCP2 negative with/without genotype	OR	CI-	CI+	p value
**DRB1*01**	218/579	142/440	1.17	0.91	1.49	0.2174
**DRB1*03**	123/674	164/418	**0.47**	**0.36**	**0.61**	**<0.0001**
**DRB1*04**	534/263	212/370	**3.54**	**2.83**	**4.43**	**<0.0001**
**DRB1*07**	72/725	60/522	0.86	0.6	1.24	0.4266
**DRB1*08**	37/760	74/508	**0.33**	**0.22**	**0.5**	**<0.0001**
**DRB1*09**	31/766	21/561	1.08	0.61	1.9	0.7865
**DRB1*10**	38/759	11/571	**2.6**	**1.32**	**5.13**	**0.0044**
**DRB1*11**	56/741	58/524	**0.68**	**0.47**	**1**	**0.05**
**DRB1*12**	26/771	25/557	0.75	0.43	1.31	0.3153
**DRB1*13**	75/722	152/430	**0.29**	**0.22**	**0.4**	**<0.0001**
**DRB1*14**	17/780	24/558	**0.51**	**0.27**	**0.95**	**0.0316**
**DRB1*15**	183/614	137/445	0.97	0.75	1.25	0.8016
**DRB1*16**	14/783	8/574	1.28	0.53	3.08	0.578
**DRB04*01**	333/464	119/463	**2.79**	**2.18**	**3.57**	**<0.0001**
**DRB04*04**	110/687	45/537	**1.91**	**1.32**	**2.75**	**0.0004**
**SE**	681/116	326/256	**4.61**	**3.57**	**5.96**	**<0.0001**

Anti-CII single positive >29AU/ml	Anti-CII positive with/without genotype	Anti-CII negative with/without genotype	OR	CI-	CI+	p value
**DRB1*01**	13/26	142/440	1.55	0.78	3.1	0.212
**DRB1*03**	18/21	164/418	**2.18**	**1.13**	**4.21**	**0.021**
**DRB1*04**	5/34	212/370	**0.26**	**0.1**	**0.67**	**0.0028**
**DRB1*07**	6/33	60/522	1.58	0.64	3.93	0.3194
**DRB1*08**	7/32	74/508	1.5	0.64	3.53	0.3474
**DRB1*09**	1/38	21/561	0.7	0.09	5.37	0.7327
**DRB1*10**	2/37	11/571	2.81	0.6	13.13	0.1715
**DRB1*11**	3/36	58/524	0.75	0.22	2.52	0.6442
**DRB1*12**	2/37	25/557	1.2	0.27	5.28	0.805
**DRB1*13**	9/30	152/430	0.85	0.39	1.83	0.6749
**DRB1*14**	1/38	24/558	0.61	0.08	4.65	0.6314

**DRB1*15**	8/31	137/445	0.84	0.38	1.87	0.6654
**DRB1*16**	1/38	8/574	1.89	0.23	15.49	0.5473
**DRB04*01**	3/36	119/463	0.32	0.098	1.07	0.0523
**DRB04*04**	2/27	45/537	0.65	0.15	2.76	0.5517
**SE**	20/19	326/256	0.83	0.43	1.58	0.5647

**Anti-CCP/anti-CII double positive**	**Anti-CII and anti-CCP2 positive with/without genotype**	**Anti-CII and anti-CCP2 negative with/without genotype**	**OR**	**CI-**	**CI+**	**p value**
**DRB1*01**	17/41	142/440	1.28	0.71	2.33	0.4091
**DRB1*03**	15/43	164/418	0.89	0.48	1.64	0.7078
**DRB1*04**	35/23	212/370	**2.66**	**1.53**	**4.61**	**0.0004**
**DRB1*07**	3/55	60/522	0.47	0.14	1.56	0.2105
**DRB1*08**	4/54	74/508	0.51	0.18	1.45	0.1965
**DRB1*09**	2/56	21/561	0.95	0.22	4.18	0.9502
**DRB1*10**	1/57	11/571	0.91	0.12	7.18	0.9292
**DRB1*11**	1/57	58/524	**0.16**	**0.02**	**1.17**	**0.0385**
**DRB1*12**	3/55	25/557	1.22	0.36	4.15	0.7555
**DRB1*13**	10/48	152/430	0.59	0.29	1.19	0.1382
**DRB1*14**	1/57	24/558	0.41	0.05	3.07	0.3684
**DRB1*15**	10/48	137/445	0.68	0.33	1.37	0.2769
**DRB1*16**	0/58	8/574	0	-	-	0.3689

Supplementary table S8. Odds ratios (OR) and 95% confidence intervals (CI) for non-specific reactivity in relation to individual HLA-DRB1* alleles in 1695 RA patients. Non-specific reactivity (n=316) was defined as > 29 AU/ml, representing the 95th percentile of healthy controls (Mullazehi M et al, Ann Rheum Dis 2007;66;537), together with higher OD values in BSA coated control wells as compared to CII coated wells. The comparative group consisted of all anti-CII negative patients (n=1379). Significant associations are depicted in bold.

	**Non-specific reactivity with/without genotype**	**Non-specific reactivity with/without genotype**	**OR**	**CI-**	**CI+**	**p value**
**DRB1*01**	77/239	360/1019	0.91	0.69	1.21	0.5239
**DRB1*03**	65/251	287/1092	0.99	0.73	1.33	0.9236
**DRB1*04**	187/129	746/633	1.23	0.96	1.58	0.1015
**DRB1*07**	31/285	132/1247	1.03	0.68	1.55	0.1695
**DRB1*08**	30/286	111/1268	1.19	0.78	1.83	0.4017
**DRB1*09**	5/311	52/1327	0.41	0.16	1.04	0.0516
**DRB1*10**	8/308	49/1330	0.71	0.33	1.5	0.3635
**DRB1*11**	33/283	114/1265	1.29	0.86	1.95	0.215
**DRB1*12**	12/304	51/1328	1.03	0.54	1.95	0.933
**DRB1*13**	44/272	227/1152	0.82	0.58	1.16	0.267
**DRB1*14**	17/299	41/1338	**1.86**	**1.04**	**3.31**	**0.0397**
**DRB1*15**	61/255	320/1059	0.79	0.58	1.07	0.134
**DRB1*16**	5/311	22/1357	0.99	0.37	2.64	0.9866
**DRB04*01**	120/196	452/927	1.26	0.97	1.62	0.078
**DRB04*04**	48/268	155/1224	1.41	0.1	2	0.0511
**SE**	236/80	1007/372	1.09	0.82	1.44	0.5473
